# Impact of a care bundle for patients with blunt chest injury (ChIP): A multicentre controlled implementation evaluation

**DOI:** 10.1371/journal.pone.0256027

**Published:** 2021-10-07

**Authors:** Kate Curtis, Sarah Kourouche, Stephen Asha, Julie Considine, Margaret Fry, Sandy Middleton, Rebecca Mitchell, Belinda Munroe, Ramon Z. Shaban, Alfa D’Amato, Clare Skinner, Glen Wiseman, Thomas Buckley

**Affiliations:** 1 Susan Wakil School of Nursing and Midwifery, Faculty of Medicine and Health, University of Sydney, Camperdown, NSW, Australia; 2 Emergency Services, Illawarra Shoalhaven Local Health District, Wollongong Hospital, Wollongong, NSW, Australia; 3 Illawarra Health and Medical Research Institute, University of Wollongong, Wollongong, NSW, Australia; 4 Emergency Department, St George Hospital, Kogarah, NSW, Australia; 5 St George Clinical School, Faculty of Medicine, University of New South Wales, Kogarah, NSW, Australia; 6 School of Nursing and Midwifery and Centre for Quality and Patient Safety Experience in the Institute for Health Transformation, Deakin University, Geelong, VIC, Australia; 7 Centre for Quality and Patient Safety Experience–Eastern Health Partnership, Box Hill, VIC, Australia; 8 Faculty of Health, University of Technology Sydney, Ultimo, NSW, Australia; 9 Northern Sydney Local Health District, Hornsby, NSW, Australia; 10 Nursing Research Institute, St Vincent’s Health Network Sydney, St Vincent’s Hospital Melbourne, Fitzroy, Australia; 11 Australian Catholic University, Sydney, NSW, Australia; 12 Australian Institute of Health Innovation, Faculty of Medicine, Health and Human Sciences, Macquarie University, Macquarie Park, NSW, Australia; 13 Marie Bashir Institute for Infectious Diseases and Biosecurity, The University of Sydney, Camperdown, NSW, Australia; 14 Division of Infectious Diseases and Sexual Health, Department of Infection Prevention and Control, Westmead Hospital and Western Sydney Local Health District, Westmead, NSW, Australia; 15 New South Wales Biocontainment Centre, Western Sydney Local Health District and New South Wales Health, Warwick Farm, NSW, Australia; 16 NSW Activity Based Funding Taskforce, NSW Ministry of Health, Sydney, Australia; 17 Emergency Department, Hornsby Ku-ring-ai Hospital, Hornsby, NSW, Australia; 18 Emergency Services, Canterbury Hospital, Campsie, NSW, Australia; John Hunter Hospital and University of Newcastle, AUSTRALIA

## Abstract

**Background:**

Blunt chest injury leads to significant morbidity and mortality. The aim of this study was to evaluate the effect of a multidisciplinary chest injury care bundle (ChIP) on patient and health service outcomes. ChIP provides guidance in three key pillars of care for blunt chest injury—respiratory support, analgesia and complication prevention. ChIP was implemented using a multi-faceted implementation plan developed using the Behaviour Change Wheel.

**Methods:**

This controlled pre-and post-test study (two intervention and two non-intervention sites) was conducted from July 2015 to June 2019. The primary outcome measures were unplanned Intensive Care Unit (ICU) admissions, non-invasive ventilation use and mortality.

**Results:**

There were 1790 patients included. The intervention sites had a 58% decrease in non-invasive ventilation use in the post- period compared to the pre-period (95% CI 0.18–0.96). ChIP was associated with 90% decreased odds of unplanned ICU admissions (95% CI 0.04–0.29) at the intervention sites compared to the control groups in the post- period. There was no significant change in mortality. There were higher odds of health service team reviews (surgical OR 6.6 (95% CI 4.61–9.45), physiotherapy OR 2.17 (95% CI 1.52–3.11), ICU doctor OR 6.13 (95% CI 3.94–9.55), ICU liaison OR 55.75 (95% CI 17.48–177.75), pain team OR 8.15 (95% CI 5.52 –-12.03), analgesia (e.g. patient controlled analgesia OR 2.6 (95% CI 1.64–3.94) and regional analgesia OR 8.8 (95% CI 3.39–22.79), incentive spirometry OR 8.3 (95% CI 4.49–15.37) and, high flow nasal oxygen OR 22.1 (95% CI 12.43–39.2) in the intervention group compared to the control group in the post- period.

**Conclusion:**

The implementation of a chest injury care bundle using behaviour change theory was associated with a sustained improvement in evidence-based practice resulting in reduced unplanned ICU admissions and non-invasive ventilation requirement.

**Trial registration:**

ANZCTR: ACTRN12618001548224, approved 17/09/2018

## Introduction

Blunt chest injury leads to significant morbidity and mortality [1]. Significant force is needed to fracture the ribs or sternum [2]. A damaged chest wall impairs normal breathing by hindering chest movement due to pain [3]. If not treated promptly with sufficient analgesia, physiotherapy and respiratory support, complications such as pneumonia and respiratory failure frequently occur, causing death, long-term pulmonary impairment [4], delayed recovery and increased resource use [5, 6]. Care protocols and pathways exist but their use is irregular, and evaluation of blunt chest injury combined interventions is required [7].

**Fig 1:**
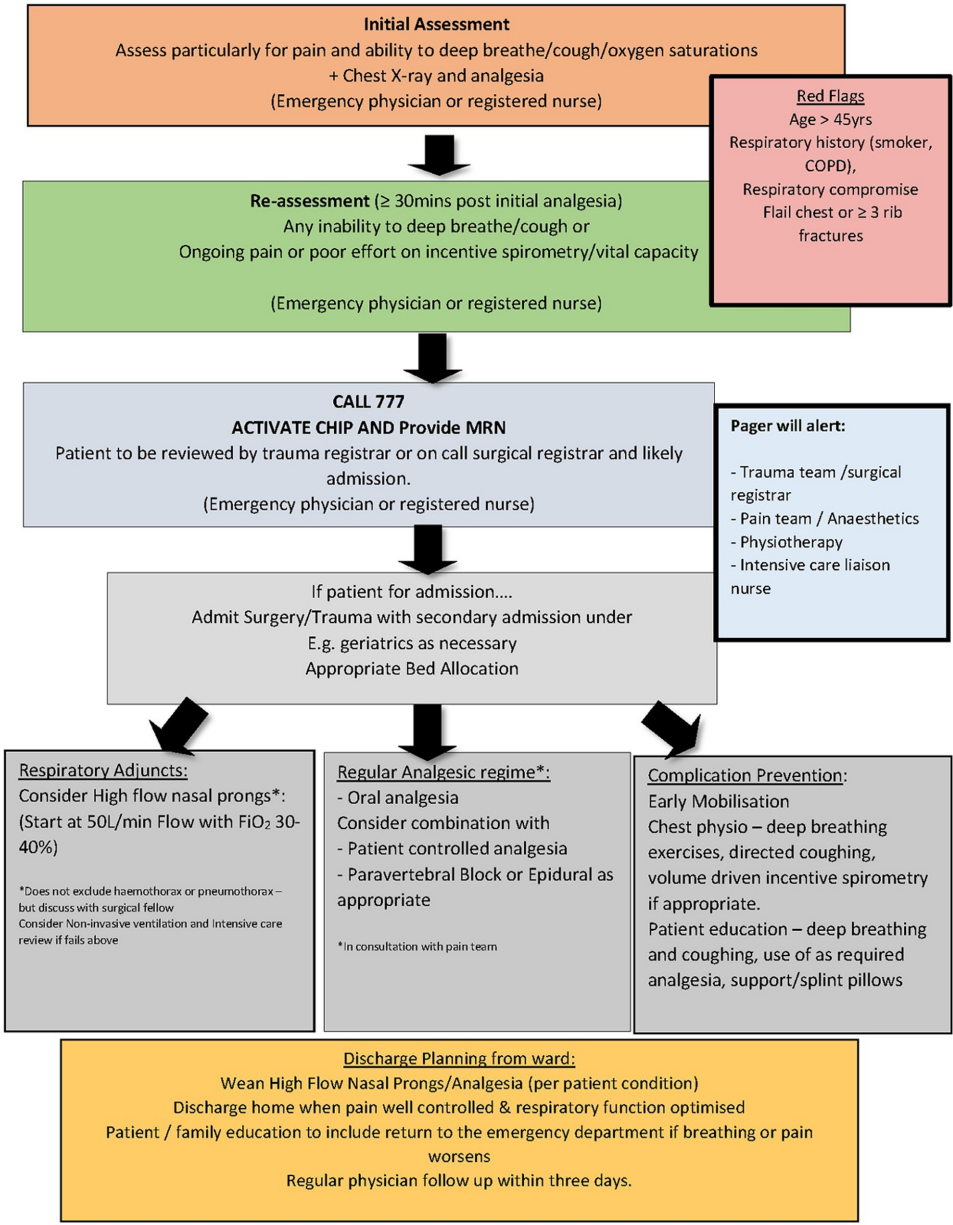
ChIP flowchart.

To address this clinical-practice gap we developed a chest injury care bundle (ChIP), consisting of an early notification system and care bundle for patients presenting through the emergency department (ED) with isolated blunt chest injury (Fig 1), through an integrative review of the literature [8]. A care bundle is a set of evidence-based interventions that when delivered together improve health outcomes more than if they were administered separately [9]. The ChIP care bundle provided guidance in three key pillars of care for patients with blunt chest injury—respiratory support, analgesia and complication prevention. ChIP was implemented with a robust and effective implementation plan [10] informed by behaviour change theory [11]. The aim of this study was to identify the effects of this multidisciplinary chest injury care bundle (ChIP) on patient and health service outcomes in two centres in regional New South Wales (NSW), Australia.

## Materials and methods

### Study population and setting

This was a controlled pre-and post-test study with two intervention and two non-intervention sites conducted between 1 July 2015–21 Nov 2017 (pre) and 22 November 2017 and 30 June 2019 (post). Patients were included in the study if they had a mechanism of injury suggesting isolated blunt chest trauma, were 18 years or older, admitted to hospital, and had either a radiological or clinical diagnosis of rib or sternum fracture. Patients were excluded if: their injury occurred while in hospital as this made the activation system not possible; had cognitive impairment rendering patients unable to participate in the care bundle; were intubated prehospital or in the ED; or had an injury requiring urgent operative intervention. The intubated and urgent operative patients were excluded as they may have received other pain management in the ICU or after operating theatre relating to the operation rather than for blunt chest injury. All blunt chest injury patients were included, not just those who met ChIP criteria due to expected implementation flow on effect into usual care [12].

The two intervention sites were two hospitals in the same local health district in regional NSW, Australia, and were matched to two hospital sites in metropolitan Sydney with similar bed numbers, staffing, case mix, resources, and chest injury case numbers according to previous years data. All four sites had ICU capability, surgical team, pain services and physiotherapists on site [13]. The two intervention sites were a 500-bed regional trauma centre treating approximately 70,000 emergency presentations annually (Site A) [14] and a 200-bed rural/regional hospital treating approximately 40,000 emergency presentations annually (Site B) [15]. ChIP was implemented at the intervention sites on the 22 November 2017.

The non-intervention sites were a 300-bed centre metropolitan centre with 36,000 presentations annually (Site C) and a 200-bed hospital with 32,000 emergency presentations annually (Site D) [16]. The non-intervention-sites continued with standard care.

Based on feasibility studies conducted in 2011 and 2014 [17, 18], a sample size of 788, with 394 patients in each arm was required to demonstrate a significant reduction in non-invasive ventilation (as an indication of respiratory deterioration) to detect a clinically important reduction in complications (10%), at alpha 0.05.

### The intervention—ChIP

Patients with blunt chest injury and ongoing pain after a single dose opioid analgesia or difficulty in coughing or taking a deep breath despite analgesia were eligible for activation of ChIP. Activation was via a paging system activated by ED clinicians (nursing or medical staff) that notified the surgical registrar, pain team, physiotherapist and intensive care unit (ICU) doctor and ICU liaison nurse. After hours, the pager notified the surgical registrar and ICU doctor. If needed overnight, the anaesthetic registrar could be contacted for pain management, as the pain service did not operate overnight. There was no physiotherapy cover overnight so the physiotherapist reviewed ChIP patients the following working day. The aged care or general medicine teams could also be contacted if required at clinician discretion per clinical need.

### Implementation of ChIP

Implementing models of care with multidisciplinary teams can be challenging as health care disciplines often have different priorities, understandings, roles and expectations about care delivery despite all having a shared goal of improving patient care [19]. A plan for the implementation of ChIP was developed using the four-step approach recommended by French et al. [20] outlined below:

Step 1. Who needed to do what differently
  The clinician behaviour to be changed was to activate and implement the ChIP care bundle [8]. ChIP required ED clinicians to identify eligible patients and activate the ChIP call, while other non-ED based clinicians responded to the ChIP activation by reviewing the patient and initiating tailored patient care guided by the ChIP protocol.
Step 2. Which barriers and enablers needed to be addressed
  To identify facilitators and barriers to ChIP implementation, a survey based on the Theoretical Domains Framework [21] was administered to almost 200 staff across 12 hospital departments [10, 22]. Nine facilitators and six barriers were identified across eight domains of the Theoretical Domains Framework and are described in detail elsewhere [10].
Step 3. What behaviour change techniques could overcome the modifiable barriers and enhance the enablers
  The identified Theoretical Domains Framework domains were mapped using the Behaviour Change Wheel to develop implementation strategies and presented in detail elsewhere [10]. Seventeen behaviour change techniques were identified (see S1 File). The implementation strategies used were: i) face-to-face educational sessions, including a video featuring local staff including managerial staff demonstrating their support: https://youtu.be/VlMz1PjzmBk; ii) audits and feedback to provide staff with data on their progress; and iii) reminders in the form of flyers, an icon prompt on the electronic medical record, and email and newsletter notification [10] (see S2 File). There was also a clinical champion at each site.
Step 4. Measurement of how well ChIP was implemented
  The fidelity, dose and reach of the implementation of ChIP, and the impact of ChIP on patient and health service outcomes. The fidelity of the implementation of ChIP was high with almost all (97.6%) of the strategies either fully (76.2%) or partially (21.4%) followed [23]. Evaluation of fidelity of ChIP delivery (i.e. whether the intervention was delivered as intended) found that ChIP was delivered to 97.1% of eligible isolated blunt chest injured patients at the intervention sites [24]. The current study investigates the impact of ChIP on all patients with blunt chest injury not just those who had a ChIP activation (to account for ‘clinical creep’ of the ChIP interventions into usual care) and health service outcomes.

### Outcome measures

The primary outcome measures were reduction in non-invasive ventilation use, unplanned ICU admissions, and mortality rates. Non-invasive ventilation (NIV) was defined as the need for NIV ventilatory support as determined by the treating medical staff. Unplanned ICU admissions were admissions to the ICU where the admission was not planned from ED. ICU stays post a planned operative procedure were excluded. Mortality was measured for patient deaths in hospital. Patients receiving planned palliative care were identified through medical records.

Secondary outcome measures of pulmonary embolism, deep vein thrombosis, delirium, and urinary tract infections were defined according to the International Statistical Classification of Disease version-10 Australian modification (ICD-10-AM) code definitions. Pneumonia was defined as radiological evidence of pulmonary air-space opacification, together with medical record documentation of a clinical diagnosis of pneumonia and treatment with antibiotics [25]. Rapid response team activations were defined as hospital responses to a deteriorating patient according to the NSW Health state policies for deteriorating patients [26].

### Data sources and collection

Patients were identified through the state database the NSW Admitted Patient Data Collection (APDC) according to preselected ICD-10-AM codes (see S3 File). Patient medical records were screened retrospectively for inclusion and data collection. The data collection period was from 01 July 2015 to 21 November 2017 (pre) and from 22 November 2017 to 30 June 2019 (post).

Once included, patient data were obtained from two sources: i) the APDC provided demographic data such as age and sex, ICU/hospital length of stay and procedures; ii) the site medical records were used to collect other clinical treatments and data relating to adherence to the ChIP protocol such as time to analgesia and administration of high flow nasal prong oxygen; unplanned admission to ICU and commencement of non-invasive ventilation. Data were collated and de-identified prior to analysis.

Clinical information included injury(s), mechanism of injury, injury date and time, injury severity score, Charlson Comorbidity Index (CCI), and whether patients received a trauma call activation. Injury data were collected according to the Abbreviated Injury Scale. The injury severity score (ISS) and the New Injury Severity Score (NISS) are internationally recognised scoring systems for the combined effects of trauma and were calculated from the abbreviated injury scores [27]. The injury severity score ranges from 1 to 75, with a score ≥15 indicating severe injuries. The NISS has been included as it may be a better predictor for blunt injury and does not discriminate for body region in the score [28].

The CCI is a scoring system that identifies and assigns weights for 17 pre-existing comorbidities based on their association with mortality [29]. Polytrauma was defined as a patient with ≥2 abbreviated injury scores ≥2 in two or more body regions. If a trauma call was activated in the ED for patients presenting with severe injuries or a high-risk mechanism of injury per local policy this was noted as an additional team response had been activated and that these patients had the potential for severe injury.

### Analysis

Statistical analyses were performed using SPSS v26 (SPSS Statistics for Windows, Version 26.0. Armonk, NY: IBM Corp). Descriptive analysis of pre- and post-period groups at both intervention and control sites. Differences in patient characteristics, clinical outcomes, and healthcare utilization pre- compared to post-period for the intervention group and control group and to compare intervention to control in the post- period. Odds ratios (OR) and 95% confidence intervals (95%CI) were calculated using marginal logistic or linear regression and ORs were adjusted for age, ISS, mechanism of injury and CCI. A p-value of <0.05 was considered statistically significant. Temporal changes for health service outcomes between groups, high flow nasal oxygen and analgesia over the research period were examined.

### Ethics and reporting

Research conducted as part of this study adhered to the National Statement on Ethical Conduct in Human Research by the Australian National Health and Medical Research Council [30], and was approved by the NSW Population & Health Services Research Ethics Committee (HREC/17/CIPHS/56). Study methods were reported in line with The Standards for Reporting Implementation Studies (STaRI) [31] (see S4 File) and the Strengthening the Reporting of Observational Studies in Epidemiology (STROBE) Statement [32] (see S5 File).

## Results

There were 1790 patients included in the final analysis. ChIP was activated for 68.4% (n = 409) of post-period presentations at intervention sites. At the invention sites, the pre- (n = 601, 50.1%) and post-period groups (n = 598, 49.9%) were similar in demographics, mode of arrival, mechanism of injury and severity of injuries sustained (Table 1).

**Table 1:** Patient characteristics intervention vs control (overall study period) (N = 1790).

					Comparisons		
	Intervention		Control		Intervention	Control	Post-period
	Pre-period	Post-period	Pre-period	Post-period	Pre vs post	Pre vs post	Intervention vs Control
	n = 601	n = 598	n = 336	n = 255			
Age, Median (IQR)	68.5 (50.2–82.7)	69.9 (54.3–82.4)	80.5 (66.2–88.1)	80.5 (65.9–87.6)	MW = 171121.5 p = 0.15	MW = 42030 p = 0.69	MW = 54045 p<0.001
Female, n %	380 (63.2%)	350 (58.5%)	152 (45.2%)	135 (52.9%)	Χ^2^_1_ = 2.59, p = 0.11	Χ^2^_1_ = 3.14, p = 0.08	Χ^2^_1_ = 2.05 p = 0.15
CCI score, median (IQR)	3 (1–6)	3 (1–5)	5 (3–6)	4 (3–6)	MW = 177606.5, p = 0.88	MW = 41523, p = 0.52	MW = 59936.5 p<0.001
COPD, n %	98 (16.3%)	85 (14.2%)	21 (6.3%)	26 (10.2%)	Χ^2^_1_ = 0.86, p = 0.35	Χ^2^_1_ = 2.57, p = 0.11	Χ^2^_1_ = 2.21 p = 0.14
Asthma, n %	55 (9.2%)	53 (8.9%)	34 (10.1%)	26 (10.2%)	Χ^2^_1_ = 005, p = 0.94	Χ^2^_1_ = 0.000, p = 1.00	Χ^2^_1_ = 0.24 p = 0.63
Other chronic lung disease	43 (7.2%)	35 (5.9%)	12 (3.6%)	11 (4.3%)	Χ^2^_1_ = 0.64, p = 0.43	Χ^2^_1_ = 0.06, p = 0.81	Χ^2^_1_ = 0.56 p = 0.46
Pneumonia on arrival, n %	41 (6.8%)	43 (7.2%)	5 (1.5%)	11 (4.3%)	Χ^2^_1_ = 0.02, p = 0.89	Χ^2^_1_ = 3,39, p = 0.07	Χ^2^_1_ = 2.03 p = 0.15
Smoker (past or current), n %	247 (41.1%)	250 (41.8%)	71 (21.1%)	64 (25.1%)	Χ^2^_1_ = 0.04, p = 0.85	Χ^2^_1_ = 1.08, p = 0.30	Χ^2^_1_ = 20.74, p<0.001
Out of hours presentation ^c^	391 (65.1%)	429 (71.7%)	240 (71.4%)	180 (70.6%)	Χ^2^_1_ = 5.88, p = 0.02	Χ^2^_1_ = 0.02, p = 0.90	Χ^2^_1_ = 0.07 p = 0.80
**Mode of arrival**					Χ^2^_2_ = 2.78, p = 0.26	Χ^2^_1_ = 2.61, p = 0.11	Χ^2^_2_ = 40.00, p<0.001
Ambulance	459 (76.6%)	480 (80.4%)	222 (66.3%)	151 (59.4%)			
Walk in	138 (23%)	116 (19.4%)	113 (33.7%)	103 (40.6%)			
Helicopter	2 (0.3%)	1 (0.2%)	0 (0%)	0 (0%)			
Fixed wing	0 (0%)	0 (0%)	0 (0%)	0 (0%)			
Transferred in	21 (3.5%)	87 (14.6%)	0 (0%)	3 (1.2%)	Χ^2^_1_ = 43.24, p<0.001	Χ^2^_1_ = 1.98, p = 0.16	Χ^2^_1_ = 32.54, p<0.001
ISS, median (IQR)	5 (2–10)	9 (3–10)	5 (2–9)	5 (2–9)	MW = 173802, p = 0.32	MW = 41123.5, p = 0.40	MW = 63396.5 p<0.001
NISS, median (IQR)	6 (2–13)	9 (3–13)	6 (2–11)	5 (2–10)	MW = 177142, p = 0.70	MW = 40828.5, p = 0.33	MW = 62689.5 p<0.001
Polytrauma^d^, n %	18 (3%)	12 (2%)	2 (0.6%)	1 (0.4%)	Χ^2^_1_ = 0.83, p = 0.36	Χ^2^_1_ = 0.000, p = 1.00	Χ^2^_1_ = 2.12, p = 0.15
Trauma call, n %	181 (30.3%)	110 (18.6%)	6 (1.8%)	1 (0.4%)	Χ^2^_1_ = 21.52, p<0.001	Χ^2^_1_ = 1.35, p = 0.25	Χ^2^_1_ = 50.00, p<0.001
**Chest injuries**							
Sternum injury, n %	68 (11.3%)	66 (11%)	26 (7.7%)	23 (9%)	Χ^2^_1_ = 0.004, p = 0.95	Χ^2^_1_ = 0.17, p = 0.68	Χ^2^_1_ = 0.58, p = 0.45
Rib Fractures	578 (96.2%)	567 (94.8%)	323 (96.1%)	239 (93.7%)	Χ^2^_1_ = 0.99, p = 0.32	Χ^2^_1_ = 1.32, p = 0.25	Χ^2^_1_ = 0.23, p = 0.64
Rib fractures <3 (includes clinical) ^e^, n %	380 (65.7%)	340 (60%)	211 (65.3%)	162 (67.8%)			
Rib fractures ≥3 ribs or flail, n %	198 (34.3%)	227 (40%)	112 (34.7%)	77 (32.2%)			
Lung injury, any, n %	158 (26.3%)	118 (19.7%)	52 (15.5%)	33 (12.9%)	Χ^2^_1_ = 6.91, p = 0.01	Χ^2^_1_ = 0.58, p = 0.45	Χ^2^_1_ = 5.20, p = 0.02
**Mechanism**					Χ^2^_2_ = 5.48, p = 0.07	Χ^2^_2_ = 2.99, p = 0.22	Χ^2^_2_ = 34.45, p<0.001
Fall ^f^, n %	323 (53.7%)	357 (59.7%)	278 (82.7%)	205 (80.4%)			
Vehicle related injury, n %	210 (34.9%)	172 (28.8%)	46 (13.7%)	33 (12.9%)			
Other mechanism ^g^, n %	68 (11.3%)	69 (11.5%)	12 (3.6%)	17 (6.7%)			

^c^ Out-of-hours presentations include weekdays 4pm-8am, weekends and public holidays.

^d^ Polytrauma = two or more abbreviated injury scores (AIS) greater than 2 in 2 or more body regions.

^e^ Clinical rib fractures include injuries where there is no documented rib fracture on imaging; however, patient has significant pain.

^f^ Fall includes standing, height and ladder.

^g^ Other mechanism includes blunt impact from objects or animals, and other mechanisms such as from cough or CPR.

Abbreviations: CCI = Charlson Comorbidity Index, ChIP = blunt chest injury care bundle protocol, COPD = Chronic obstructive pulmonary Disease, ISS = injury severity score, IQR = interquartile range, NISS = New injury severity score, MW = Mann-Whitney.

Note–for all 2 x 2 analysis–chi-square with continuity correction reported.

The pre-period group had a higher proportion of trauma calls despite no difference to mechanism or severity of injury (ISS/NISS, p < 0.01). At control sites (n = 591), there were no differences pre- and post- the implementation date.

In the post-period, patients at the intervention sites (n = 1199) were younger, had a lower CCI, though there were similar frequencies of asthma, COPD and other chronic lung conditions compared to the control hospitals (n = 691). The patients at intervention sites had less falls and higher ISS/NISS scores compared to control sites in the post-period. The injuries were similar across sites; however, internal lung injuries were more common at intervention sites.

### Clinical and health service outcomes

Intervention site patients had a 58% decrease in NIV use in the post- compared to the pre-period (OR 0.42; 95% CI 0.18–0.96) (Table 2). In the post-period, no difference was seen between intervention and control sites for NIV use. Hospital length of stay increased by 1 day at intervention sites post-period compared to pre-period (p < 0.001).

**Table 2:** Clinical outcomes and healthcare utilisation for intervention and control hospitals.

	Intervention			Control			Post only	
	n %			n %			Intervention vs Control	
	Pre-period	Post-period	Odds ratio (95%CI)	Pre-period	Post-period	Odds ratio (95%CI)/median (IQR)	Odds ratio (95%CI)/median (IQR)	Adjusted Odds ratio (95%CI)^a^
**Patient Outcomes**								
Non-Invasive Ventilation	19 (3.2%)	8 (1.3%)	0.42 (0.18–0.96)*	2 (0.6%)	4 (1.6%)	2.66 (0.48–14.65)	0.85 (0.25–2.85)	0.93 (0.27–3.29)
Unplanned ICU admissions	19 (3.2%)	6 (1%)	0.31 (0.12–0.78)*	21 (6.3%)	18 (7.1%)	1.14 (0.59–2.19)	0.13 (0.05–0.34)	0.10 (0.04–0.29)*
Mortality	9 (1.5%)	11 (1.8%)	1.23 (0.51–2.99)	2 (0.6%)	4 (1.6%)	2.66 (0.48–14.65)	1.18 (0.37–3.73)	1.76 (0.51–6.15)
Palliative	7 (77.8%)	10 (90.9%)	2.86 (0.22–37.99)	2 (100%)^b^	3 (75%)		3.33 (0.16–70.91)	4.55 (0.04–581.56)
Pneumonia	43 (7.2%)	28 (4.7%)	0.64 (0.39–1.04)	18 (5.4%)	18 (7.1%)	1.34 (0.68–2.63)	0.65 (0.35–1.19)	0.75 (0.39–1.45)
Mechanical ventilation	2 (0.3%)	0 (0%)		0 (0%)	0 (0%)			
Deep vein thrombosis	1 (0.2%)	1 (0.2%)		1 (0.3%)	0 (0%)			
Delirium	39 (6.5%)	34 (5.7%)	0.87 (0.54–1.40)	30 (8.9%)	30 (11.8%)	1.36 (0.8–2.32)	0.45 (0.27–0.76)*	0.63 (0.36–1.09)
Pulmonary Embolism	3 (0.5%)	1 (0.2%)		0 (0%)	1 (0.4%)			
Urinary tract infection	31 (5.2%)	18 (3%)	0.57 (0.32–1.03)	20 (6%)	30 (11.8%)	2.11 (1.17–3.8)*	0.23 (0.13–0.43)*	0.27 (0.14–0.52)*
**HS outcomes**								
LOS, days median (IQR)	3 (1–7)	4 (2–7)	MW = 154363, p<0.001	4 (1–7)	3 (1–7)	MW = 42049.5, p = 0.70	MW = 70310.5, p = 0.07	
ICU LOS for unplanned admission, hours median (IQR)	2 (0–53)	41 (0–100)	MW = 44 p = 0.44	6 (0–27)	17 (0–45)	MW = 172.5, p = 0.65	MW = 43, p = 0.50	
Activation of hospital rapid response team (RTT) for clinical deterioration	89 (14.8%)	111 (18.6%)	1.31 (0.97–1.78)	25 (7.4%)	43 (16.9%)	2.52 (1.5–4.26)*	1.12 (0.76–1.66)	1.4 (0.93–2.12)

^a^ Adjusted for age, CCI, ISS and MOI.

*significant P <0.05.

^b^ both the deaths were palliative.

Unplanned ICU admissions were 90% less likely at the intervention sites compared to the control groups in the post-period (OR 0.1; 95% CI 0.04–0.29). There was a 69% decrease in unplanned ICU admissions in the post- compared to the pre-period group at the intervention sites compared to no change in the control groups (OR 0.31; 95% CI 0.12–0.78). There were no differences in unplanned ICU length of stay.

In the post-period, patients at control sites were 73% more likely to have urinary tract infection (OR 0.27; 95% CI 0.14–0.52) compared to intervention sites. There were no differences identified for mortality (including palliated and non-palliated patients), delirium, pneumonia, mechanical ventilation, deep vein thrombosis, nor pulmonary embolism.

There was no significant difference between control and intervention sites for activation of rapid response teams following ChIP implementation. However, activation of rapid response teams increased by OR of 2.5 at the control sites (95%CI 1.5–4.26), whereas they remained stable at the intervention site during pre- and post-period (OR 1.31; 95% CI 0.97–1.78).

Length of hospital stay was not significantly different between intervention and control groups though at intervention sites post-period was longer by 1 day compared to pre-period (p < 0.001).

### Clinical care

At intervention sites, the post-period group had higher odds of health service reviews (surgical, physiotherapy, ICU, and pain team) compared to pre-period patients (Table 3). At the control sites, surgical reviews were 31% lower odds in the post-period group OR 0.69 (95% CI 0.49–0.98) but OR for physiotherapy reviews were 1.99 times higher odds (95% CI 1.42–2.78) than the pre-period group. Patients were more likely to receive high flow nasal oxygen, and patient education post-period at both intervention and control sites compared to pre-period.

**Table 3:** Intervention delivery comparison between intervention and control.

	Intervention			Control			Post only	
	n %			n %			Intervention vs Control	
	Pre-period	Post-period	Odds ratio (95%CI)/median (IQR)	Pre-period	Post-period	Odds ratio (95%CI)/median (IQR)	Odds ratio (95%CI)/median (IQR)	Adjusted Odds Ratio (95%CI)^a^
**Reviews**								
Surgical review	352 (58.6%)	466 (77.9%)	2.50 (1.94–3.21)*	135 (40.2%)	81 (31.8%)	0.69 (0.49–0.98)*	7.58 (5.47–10.52)*	6.6 (4.61–9.45)*
Physiotherapy review	352 (58.7%)	470 (79%)	2.65 (2.05–3.42)*	162 (48.2%)	165 (65%)	1.99 (1.42–2.78)*	2.03 (1.47–2.81)*	2.17 (1.52–3.11)*
ICU doctor review	105 (17.6%)	268 (45.3%)	3.90 (2.99–5.08)*	47 (14%)	28 (11.1%)	0.77 (0.46–1.26)	6.67 (4.36–10.2)*	6.13 (3.94–9.55)*
ICU liaison review	38 (6.4%)	238 (40.4%)	9.96 (6.90–14.38)*	7 (2.1%)	3 (1.2%)	0.58 (0.15–2.25)	55.38 (17.53–174.94)*	55.75 (17.48–177.75)*
Pain team review	173 (28.9%)	388 (65.3%)	4.63 (3.62–5.91)*	65 (19.3%)	48 (19%)	0.98 (0.65–1.48)	8.04 (5.63–11.49)*	8.15 (5.52–12.03)*
**Analgesia**								
Analgesia plan (regular, as required, regional, IV or PCA)	509 (84.7%)	563 (94.1%)	2.91 (1.93–4.37)*	311 (92.6%)	242 (94.9%)	1.5 (0.75–2.99)	0.86 (0.45–1.66)	0.71 (0.36–1.41)
Regular analgesia charted day 1	417 (69.4%)	498 (83.6%)	2.24 (1.7–2.96)*	272 (81.2%)	209 (82%)	1.05 (0.69–1.6)	1.12 (0.76–1.65)	1.01 (0.67–1.52)
As required (PRN) Analgesia charted	443 (73.7%)	475 (79.4%)	1.38 (1.05–1.8)*	292 (87.2%)	231 (90.6%)	1.42 (0.84–2.4)	0.4 (0.25–0.64)*	0.39 (0.24–0.62)
Regional analgesia	17 (4.7%)	57 (13%)	3.06 (1.75–5.37)*	1 (0.3%)	5 (2%)	6.68 (0.78–57.54)	7.48 (2.96–18.92)*	8.79 (3.39–22.79)*
Patient controlled analgesia (PCA) charted	145 (24.1%)	198 (33.1%)	1.56 (1.21–2)*	41 (12.2%)	31 (12.2%)	1 (0.61–1.64)	3.56 (2.36–5.38)*	2.55 (1.64–3.94)*
**Other interventions**								
High flow nasal cannula	99 (16.6%)	323 (54.0%)	5.91 (4.52–7.73)*	4 (1.2%)	15 (5.9%)	5.19 (1.7–15.83)*	18.79 (10.89–32.44)*	22.07 (12.43–39.2)*
Incentive spirometry	105 (17.5%)	185 (31.6%)	2.17 (1.65–2.85)*	67 (19.9%)	12 (4.7%)	0.2 (0.11–0.38)*	9.3 (5.08–17.04)*	8.31 (4.49–15.37)*
Education	247 (41.1%)	461 (77.1%)	4.82 (3.75–6.2)*	173 (51.5%)	177 (69.7%)	2.17 (1.54–3.05)*	1.46 (1.05–2.03)*	1.4 (0.99–1.98)

^a^ Adjusted for age, CCI, ISS and MOI.

*P <0.05 significant.

In the post-period, patients at the intervention sites had higher odds between 2 and 8 of receiving health service reviews compared to patients at control sites. Intervention site patients had 2.6 (95% CI 1.64–3.94) times odds of getting a patient-controlled analgesia during their stay and 8.8 (95% CI 3.39–22.79) times odds of receiving regional analgesia compared to the control sites in the post-period. There was no difference in analgesia provision at control sites.

In the post-period, there were 22.1 higher odds for high flow nasal oxygen use (95% CI 12.43–39.2), and 8.3 (95% CI 4.49–15.37) higher odds for incentive spirometry between intervention and control groups. There was no difference for education delivery for the intervention group compared to the control group in the post-period(OR 1.4; 95% CI 0.99–1.98).

### Sustainability

**Fig 2:**
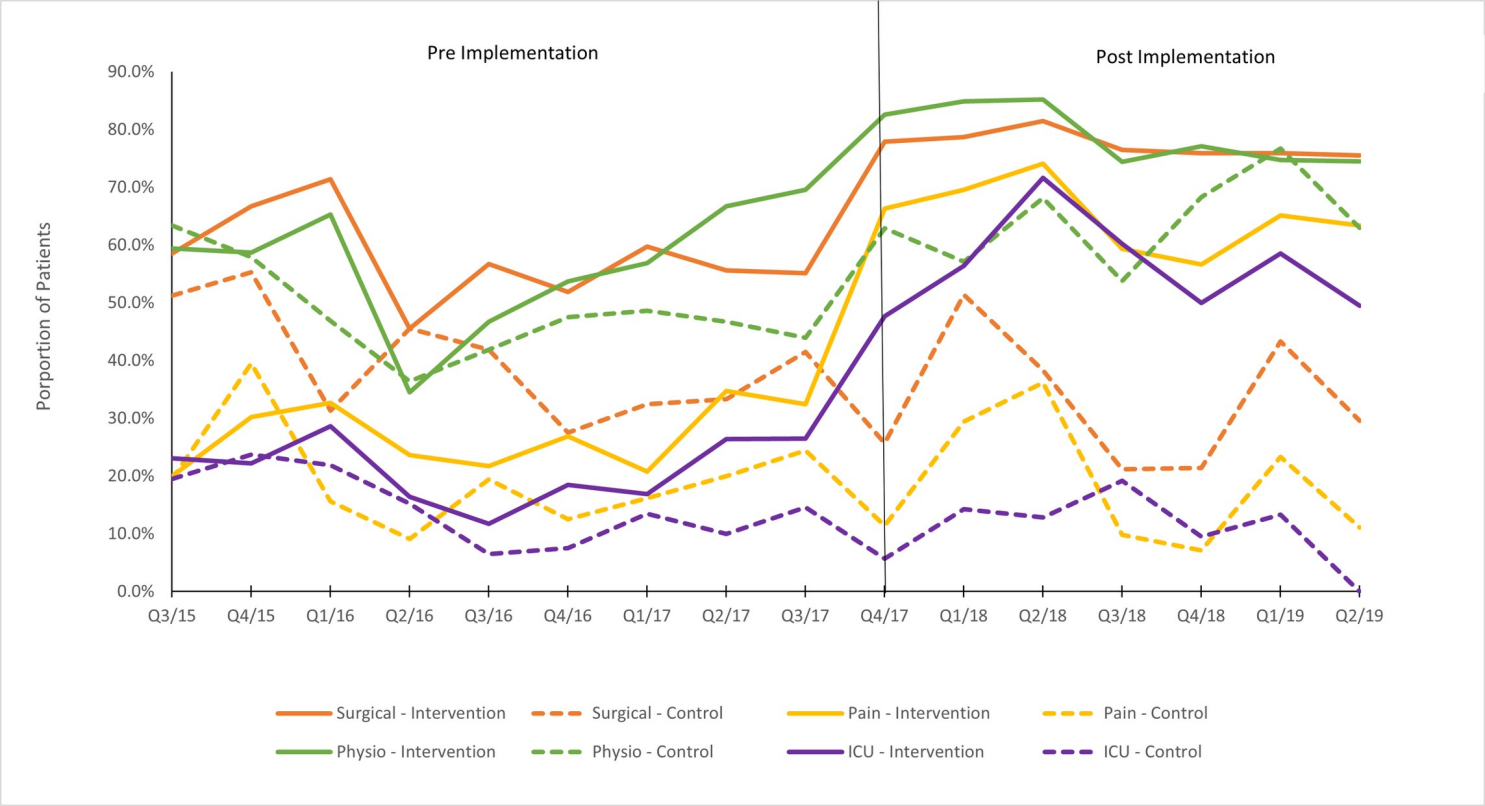
Reviews pre- and post- ChIP implementation by treatment groups [for all patients]–quarterly.

Temporal analysis demonstrated an increase in health service reviews (surgical, physiotherapy, ICU team, and pain team) at intervention sites over time, which was sustained over the post-period with a slight increase in the months leading up to implementation (Fig 2). There were no changes in specialist review use or processes at the control sites. At the intervention sites, high flow nasal oxygen and regional analgesia use increased over time and was sustained over the 19-month post-period. There were no changes in the use of high flow nasal oxygen and regional analgesia at the control sites (see S6 File).

## Discussion

Implementation of ChIP was associated with reduced NIV use and reduced unplanned ICU admissions in patients with blunt chest injury. There were no changes in mortality and the secondary outcomes of pneumonia, delirium, deep vein thrombosis or pulmonary embolism. Specialist reviews and evidence-based practices increased and were sustained over the 19-month post- period in the intervention sites. There were some increases in use in the quarter leading up to implementation date, which can be explained by the training and stakeholder meetings that were occurred in the lead up to implementation.

The findings from this study are similar to evaluations of protocols for blunt chest injury previously reported. However, previous studies only examined patients 65 years or older [33, 34] and those with three or more rib fractures [6, 33, 34]. A more recent study found reduced hospital length of stay and reduced pulmonary complications with bundled interventions (i.e. analgesia, multidisciplinary reviews, imaging and surgical intervention) delivered through a computerised clinical decision support intervention for blunt chest injury [35].

Though improvements have been made in using implementation science in the emergency and critical care context, most studies have investigated facilitators and barriers rather than conducting an evaluation of the implementation [36]. There are many instances of less than adequate implementation results in the ED setting where clinician behaviour change is difficult to achieve [37–40]. Successful implementation needs planning and strategies that address the complexity and politics of health care systems, individual practitioners, managers, context as well as strong organisational support and patronage which is influential to normalise a new practice among staff [19]

### Methodological considerations and limitations

A strength of this study is the quality of implementation using implementation science principles, including a theoretically informed intervention, proven implementation strategies targeting identified barriers and facilitators. Further, the implementation included plans for the sustainability of ChIP long-term which has been reflected in the long-term maintenance of ChIP. Implementation evaluation of new protocols and guidelines is important to consider in the effectiveness of chest injury protocols [7]. Only one other study reported using implementation science principles for to evaluate the implementation of an intervention for blunt chest injury, reporting high adherence and resulting in decreased hospital length of stay [35] with one other study reporting low adherence without the use of implementation theory [33]. Another strength is the rigour of the evaluation examining fidelity, which is not often done.

There were limitations to this study. The study was not randomised as this was a proof-of-concept study with limited funding, hence, a multisite controlled pre–post-trial was used as a pragmatic solution. Identification of patients for inclusion relied on the accuracy of ICD-10-AM coding, which can be problematic. The authors acknowledge that though the odds ratios were large, the effect sizes were small for NIV. The hospitals were matched with available presentation data prior to commencement of the study; however, the hospitals in the intervention group had higher numbers of patients with chest injury. It is possible that these sites simply saw more chest injury than the control sites given geographic location and population (regional vs metro).

ChIP relies on clinical decisions in real-time; therefore, without very clear documentation it can be difficult to ascertain if a patient meets criteria post-hoc. Therefore, all patients with chest injury were included in the analysis rather than only those with documented pain on breathing and ongoing pain after analgesia. A sub-analysis of patients meeting ChIP criteria (ongoing pain after analgesia or difficulty in coughing or taking a deep breath despite analgesia) was attempted; however, patient numbers in the pre-period group and the control groups were limited and the sub-analysis therefore was not feasible.

A large proportion (70%) of patients presented out of hours (after 4pm or on a weekend). Although the intervention was designed to ensure targeted patient care 24/7, sub analysis of the outcomes of this group was not conducted.

There were no prespecified criteria for ChIP patient transfer to ICU. This decision was at clinician discretion. A small element of clinician bias amongst the few familiar with the study at the intervention sites is plausible, however, the lack of significant change in the incidence of patient deterioration does not infer this.

## Conclusion

The results from this study have demonstrated that ChIP is a safe and effective option to reduce complications in patients with blunt chest injury. Further, it has demonstrated the importance a well-developed implementation strategy for long-term sustainable success. Upscale and spread with rigorous evaluation, including a cost-benefit analysis, should be considered with a Phase 4 translational trial of ChIP.

The high-fidelity theory-based implementation of a chest injury care bundle was associated with significant improvement in evidence-based practice resulting in sustained change in clinical practice and reductions in unplanned ICU admissions and NIV, indicating fewer patient complications. ChIP should be translated more widely for treatment of blunt chest injury patients.

## Supporting information

S1 File Implementation plan (redacted). (PDF)

S2 File Implementation resources. (PDF)

S3 File International Statistical Classification of Disease version-10 Australian modification (ICD-10-AM) codes. (PDF)

S4 File The Standards for Reporting Implementation Studies (STaRI). (PDF)

S5 File The Strengthening the Reporting of Observational Studies in Epidemiology (STROBE) Statement. (PDF)

S6 File High flow nasal prongs and analgesia within 24 hours pre- and post—ChIP implementation by treatment groups [for all patients]–quarterly. (PDF)
