# Supplementary material for: Impact of a care bundle for patients with blunt chest injury (ChIP): A multicentre controlled implementation evaluation
Source: PLoS One. 2021 Oct 7;16(10):e0256027. doi: 10.1371/journal.pone.0256027 (PMC8496821; doi:10.1371/journal.pone.0256027)
Supplement: S2 File — (PDF) [file pone.0256027.s002.pdf]

Contents:

1. ED flyer
2. Patient information sheet
3. Video Story board
4. Example of email for multidisciplinary teams
5. Newsletter advertisement
6. Presentation slides

Help Yourself to some ChIP

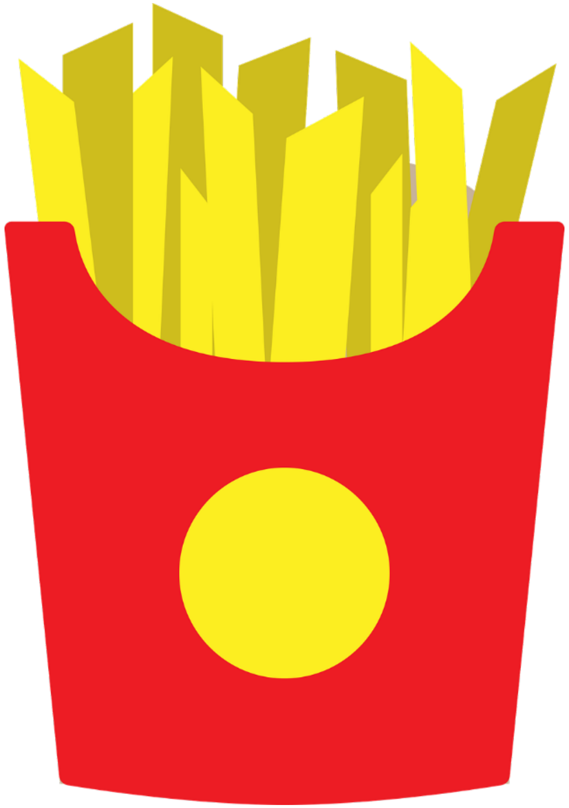

The Chest Injury Protocol (ChIP) bundle of care is like a trauma call or a stroke page - but for patients with clinical or radiological rib fractures.

ChIP is a way to let the right people know about the patient sooner, and, provides an evidenced-based guide for clinical management for analgesia, oxygenation and general care.

ChIP decreases pneumonia by 56%.

Any questions contact Kate Curtis (ED CNC)

ChIP – Go Live November 22nd!

Image by *Tomislav Kaučić* from *Pixabay*

ChIP – Information for patients

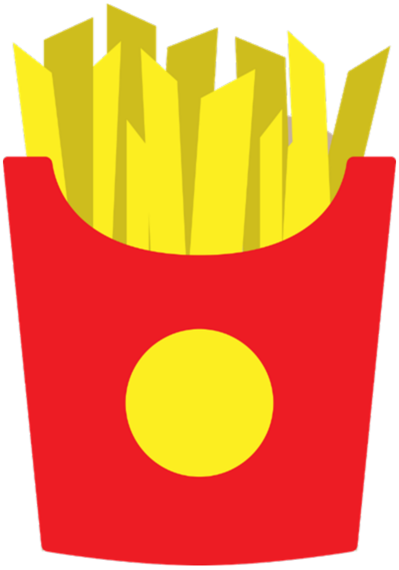

What is ChIP?

The Chest Injury Protocol (ChIP) is for patients who have sustained a blunt injury to their chest. They may or may not have confirmed rib fractures, but may have pain in the chest area as a result of trauma.

Why ChIP?

ChIP is a way to let the right staff know about the patient sooner, like physiotherapy and the pain team. Studies have shown that good pain relief, oxygenation and breathing exercises will result in less pneumonia and other complications.

How can patients get involved in ChIP?

1. Ask for a patient handout on rib fractures
2. Let a staff member know when you have pain. Especially if you can't take a deep breath or cough
3. Breathing exercises as recommended by your physio

The healthy ChIP!

Image by Tomislav Kaučić from Pixabay

Video Story board

1	2	3	4	5
ChIP animation<br>Animation 1a: protocol image	Clip:<br>“CHEST” sign<br>Consultant – ED	Clip:<br>“INJURY” sign<br>Consultant – Surgery	Clip:<br>“PATHWAY” sign<br>Consultant – ICU	Clip:<br>“For isolated clinical or radiological rib fractures”<br>Animation 2b
6	7	8	9	10
Clip:<br>↓ Pneumonia by 56%<br>ED nurses	Animation 3a/3b:<br>How does it work?	Clip: Initial assessment<br>Mock patient presents to ED holding chest, RN asking if can deep breathe and cough, patient tries and can’t.	Clip: RN talks to MO, gets analgesia charted	Clip: RN gives analgesia to patient
11	12	13	14	15
Animation:<br>Clock showing 30 min elapses	Clip: Reassessment of patient still in pain, can’t deep breathe	Clip: RN discusses with MO – “let’s activate ChIP”	Clip: Dialling emergency number	ChIP MRN<br>Switchboard<br>Page vibrating<br>eMR icon
16	17	18	19	20
Animation<br>Within 60 min	Each of the teams with signs:<br>Surg<br>Pain<br>Physio<br>ICU reg<br>ICU liaison<br>ASET	Animation:<br>After hours	Clips: Teams responding	Animation<br>ChIP bundle of care Tailored for patient needs
21	22	23	24	25
Breathing<br>Oxygen consider HFNP<br>Triflow<br>Protocol<br>Splint	Analgesia	Admit surgery and if needed<br>Aged care<br>Gen Med<br>ICU	Animation:<br>ChIP - it works	Patient Photo

Dear surgical colleagues,

We are nearing the roll out of the blunt chest injury notification protocol (ChIP) with Go Live date 22 November. Hopefully, you have already heard that ChIP was coming, some more information is enclosed in this email.

What is it?

- The ChIP bundle of care is like a trauma call or a stroke page - but for patients with clinical or radiological rib fractures

- ChIP is a way to let the right people know about the patient sooner, and, provide a guide for clinical management

Why ChIP?

- ChIP reduces the odds of blunt chest injured patients developing pneumonia by 56%.

What is your role?

As surgical team member, you may receive a page with message “ChIP” and the MRN of the patient. Where possible, the patient should be assessed within 60 minutes of the page going out.

Patients should be assessed for likely admission. They may need further reviews by ICU or Aged care. They can be admitted under a surgical team on call with a second specialty as AMO2 if required.

Patients should receive the bundle of care as per the policy

More info>

Please watch our video here.

For more information you can also refer to the full policy link

CHEST INJURY PROTOCOL - BUNDLE OF CARE

CHIP

PROF KATE CURTIS

2017

WHY?

- 89YO MALE, FALL STAIRS, INTOXICATED
- # R RIBS 1-5 WITH FLAIL SEGMENT, CONSOLIDATION RLL, CHI, SKIN TEARS
- PMHX: COPD, HT, PREV ICU ADMISSION PNEUMONIA X 2
- OBS: RR 24BPM, SPO2 95% RA, HD STABLE

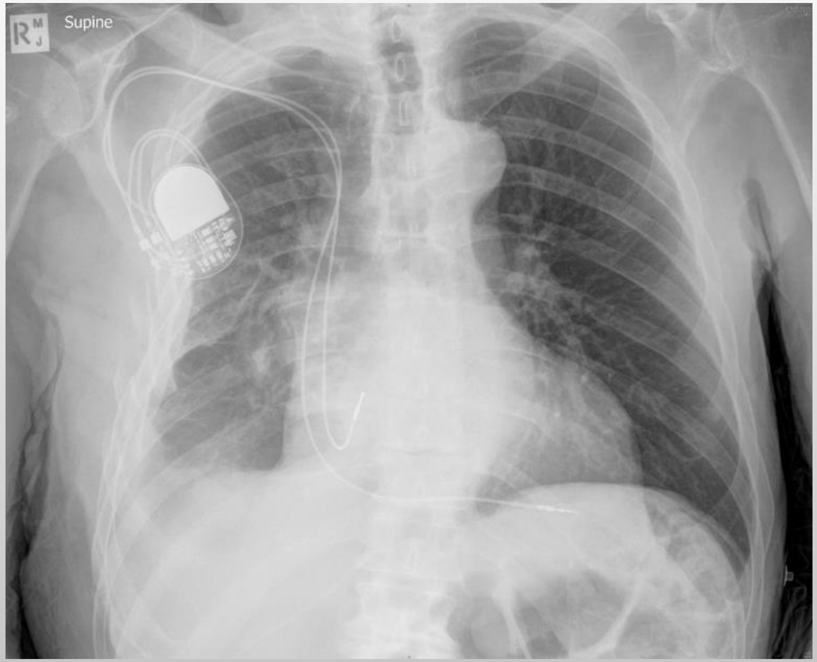

EARLY NOTIFICATION
TAILORED PATIENT CARE
EVIDENCE BASED CARE

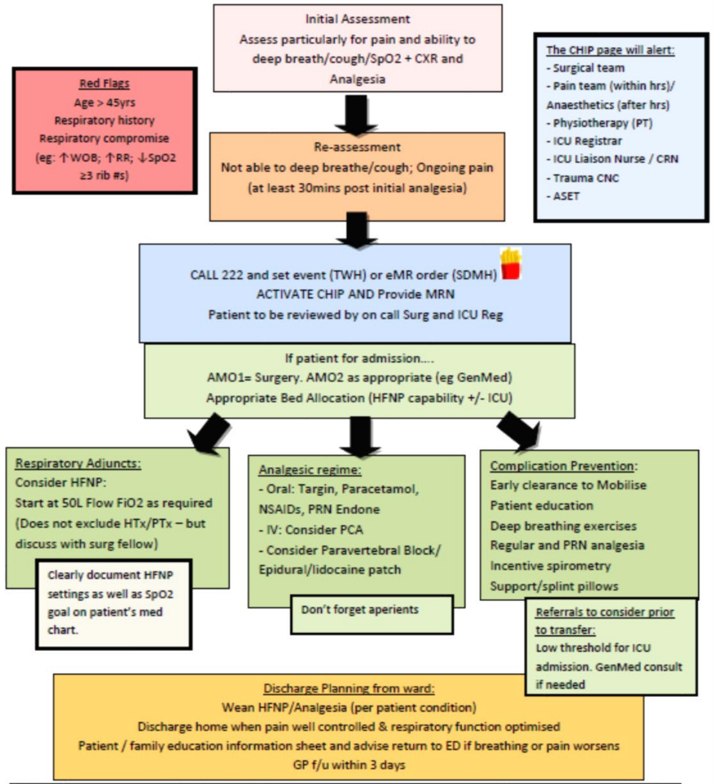

INTERNAL ONLY

ISLHD POLICY

CHIP: Early notification Chest Injury Pathway — ED CLIN PD 08

Revision 0 | Trim No. DT17/145935 | October 2017 | Page 1 of 1

THIS DOCUMENT BECOMES UNCONTROLLED WHEN PRINTED OR DOWNLOADED UNLESS REGISTERED BY LOCAL DOCUMENT CONTROL PROCEDURES

INTERNAL ONLY

RESULTS: HOSPITAL TREATMENT BEFORE-CHIP / AFTER-CHIP

Characteristic	Before-ChIP Cohort n=273	After-ChIP Cohort n=273	p value
Pain Team Review, n (%, 95%CI)	36 (13.2, 8.9-17.5)	87 (31.9, 26–37.8)	<0.001
Median time to pain team review (hours) (IQR)	30 (20–45)	27 (19–45)	0.77
Physiotherapy review, n (%, 95%CI)	235 (86.1, 81.7–90.5)	255 (93.4, 90.3–96.5)	0.01
Median time to physiotherapy review (hours) (IQR)	24 (18–41)	20 (7–28)	<0.001
Trauma team review, n (%, 95%CI)	107 (39.2, 33–45.4)	258 (94.5, 91.6–97.4)	<0.001
PCA used, n (%, 95%CI)	28 (10.3, 6.4–14.1)	44 (16.1, 11.5–20.8)	0.04
HFNP used, n (%, 95%CI)	28 (10.3, 6.4–14.1)	116 (42.5, 36.2–48.7)	<0.001
ChIP: Chest Injury Protocol; CI: Confidence Interval; IQR: Inter-quartile range; PCA: Patient Controlled Analgesia; HFNP: High flow nasal prong oxygen			

RESULTS: BEFORE-CHIP / AFTER-CHIP COHORTS

Characteristic	Before-ChIP Cohort n=273	After-ChIP Cohort n=273	p value
	Median (IQR)	Median (IQR)	
Hospital LOS (days)	4 (2–8)	4 (2–8)	0.50
	n(%, 95% CI)	n(%, 95% CI)	
Pneumonia	25 (9.2, 5.5–12.8)	12 (4.4, 1.8–7.0)	**0.03**
DVT	0	0	—
PE	0	0	—
Ventilator Support Required (CPAP/BiPAP/intubation)	6 (2.2, 0.3–4.0)	3 (1.1, -0.2–2.4)	0.50*
Death	6 (2.2, 0.3–4.0)	2 (0.7, -0.3 – 1.8)	0.30*

* Fischer's exact test used ChIP: Chest Injury Protocol; IQR: Inter-quartile range; LOS: Length of stay; DVT: Deep venous thrombosis; PE: Pulmonary embolism; CPAP: Continuous positive airway pressure; BiPAP: Bi-level positive airway pressure

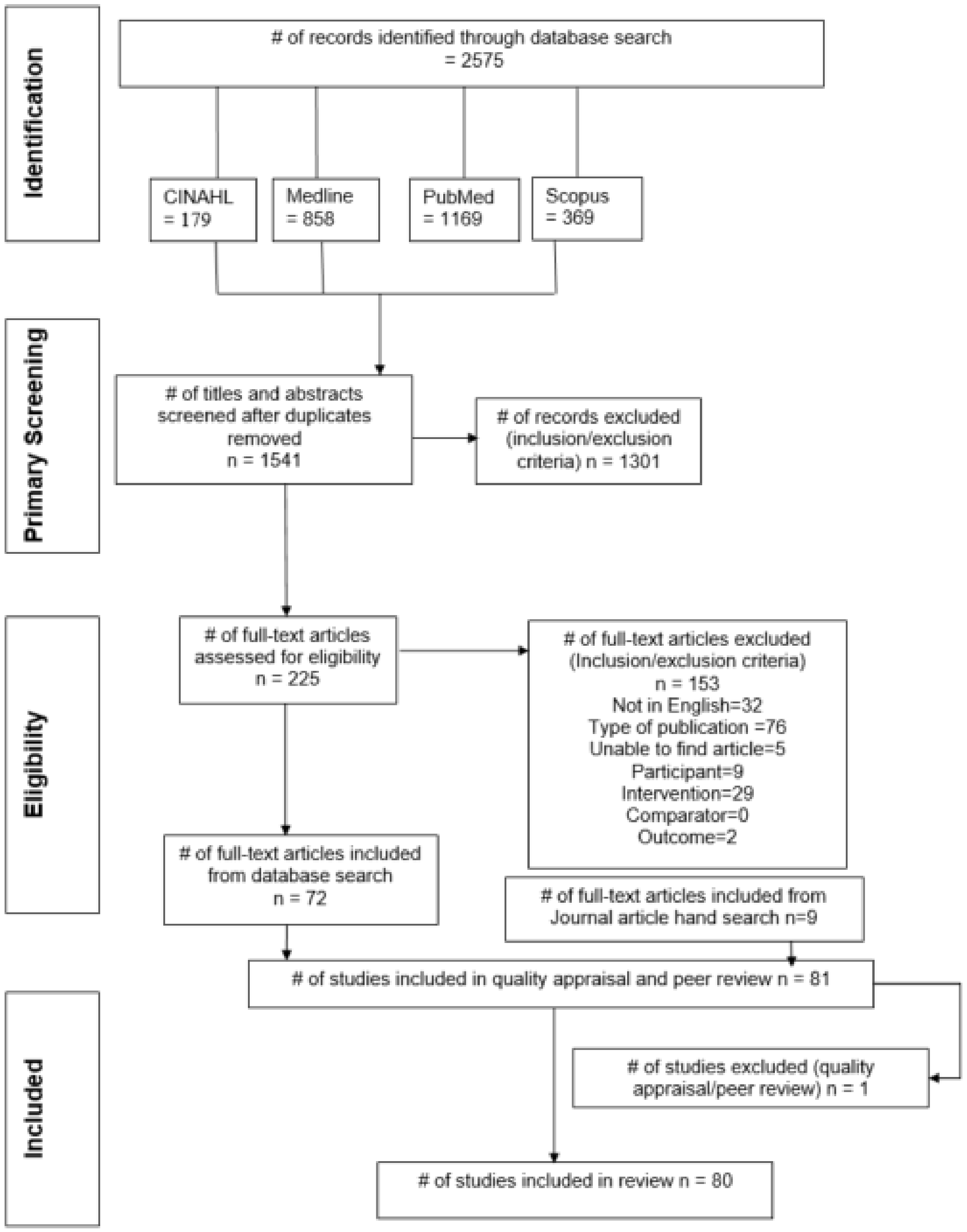

PRISMA DIAGRAM
Summary of search strategy and screening process

RESPIRATORY

HFNP

- SIMILAR EFFECTS TO CPAP,
- DECREASED LOS WHEN STARTED EARLIER
- DECREASED MORTALITY BY 9%,
- DECREASED PNEUMONIA 13-56% AS PART OF PROTOCOL
- MORE COMFORTABLE COMPARED TO CPAP

(CURTIS ET AL., 2016; HALUB ET AL., 2016; NYLAND ET AL., 2016)

CHEST PHYSIOTHERAPY

- SPLINTING
- INCENTIVE SPIROMETRY
- ACTIVE CONTROLLED BREATHING TECHNIQUES REDUCE PAIN

(BUTTS ET AL., 2017; CARRIE ET AL., 2017; CARVER, MILIA, SOMBERG, BRASEL, & PAUL, 2015; GRAMMATOPOULOU ET AL., 2010; MEYNAERTS ET AL., 1999)

Photo by Bob Osias on Unsplash

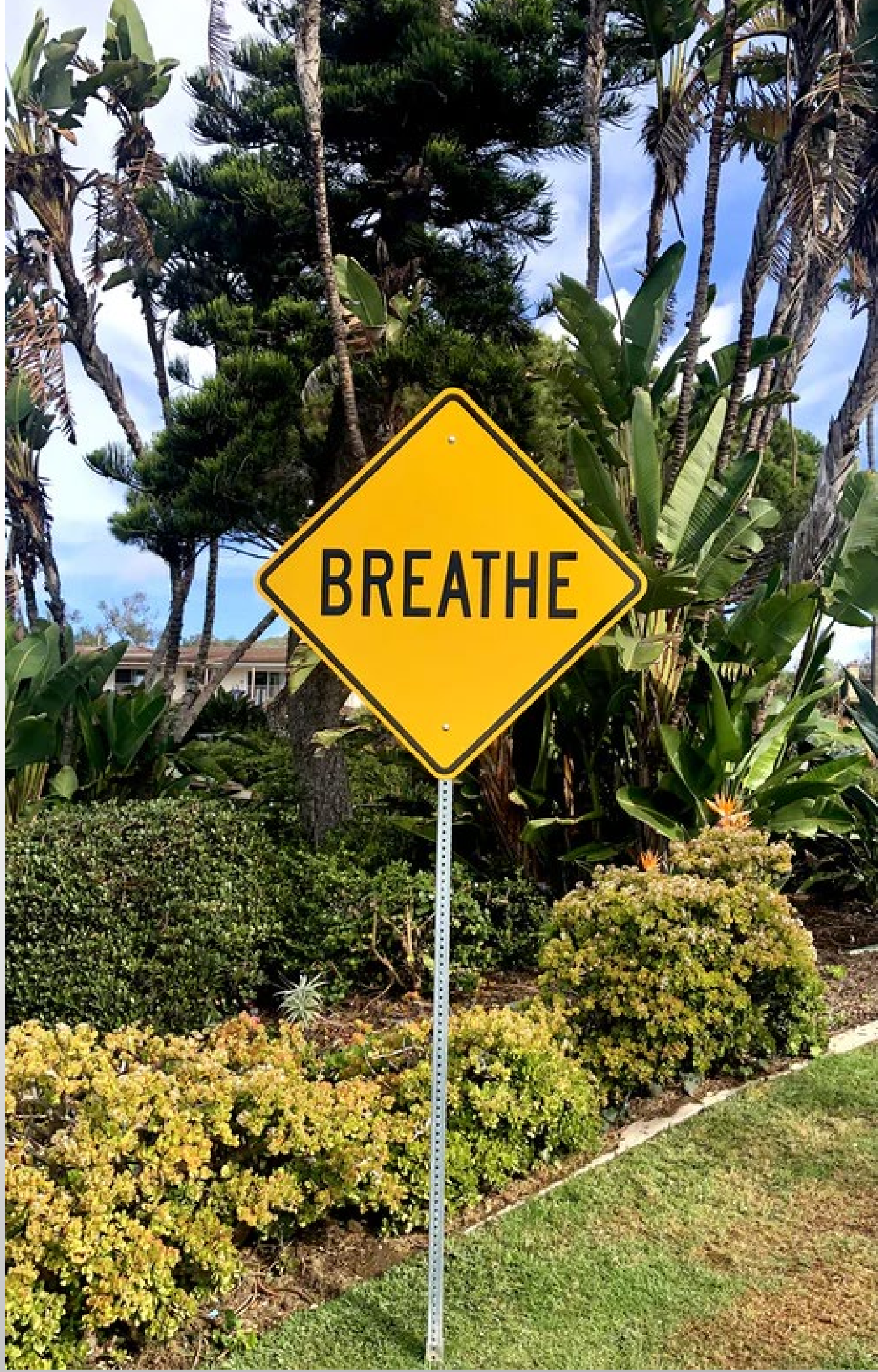

Transition from low flow to Optiflow

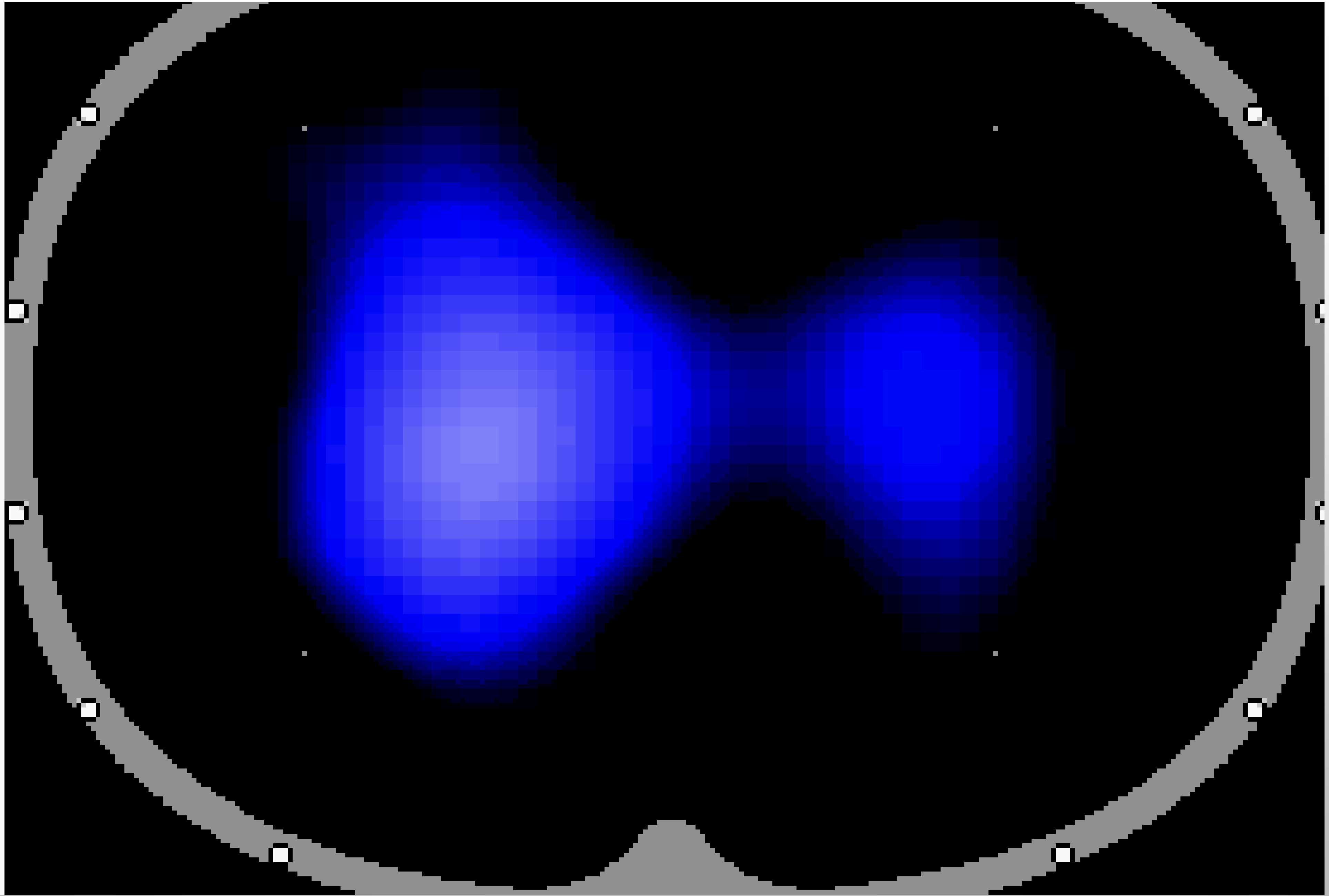

OXYGEN / HFNP DOCUMENTATION

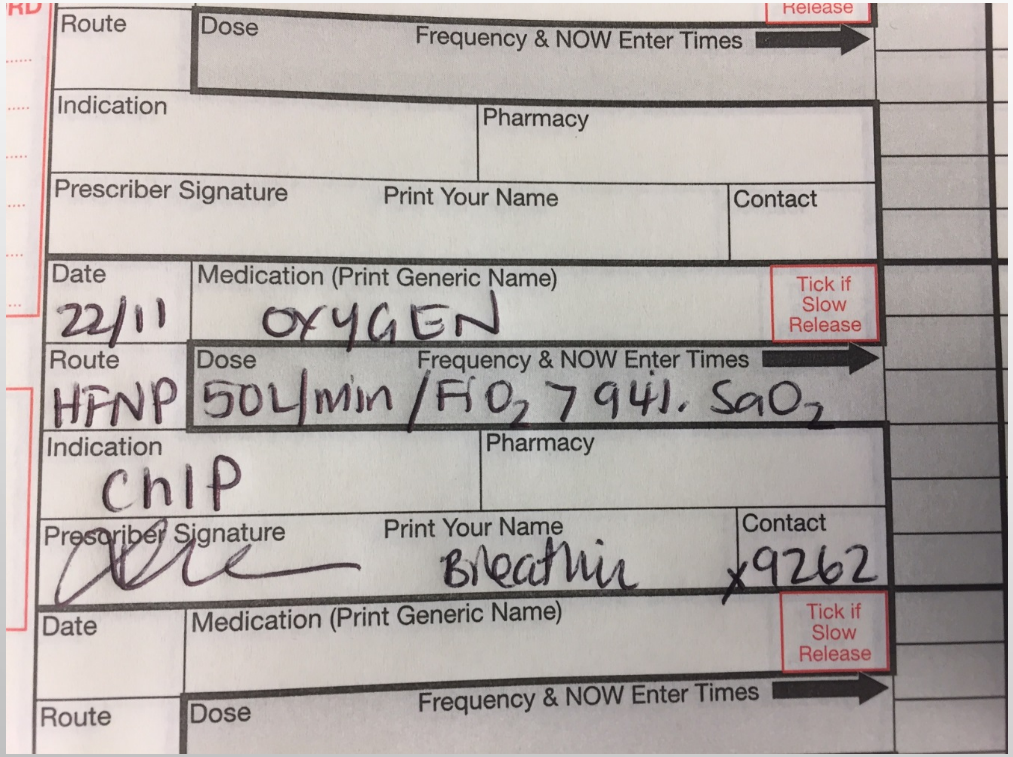

- MEDICATION CHART
- FLOW 50L/MIN
- TITRATE FIO2   TO SAO2 94-98%

If oxygen therapy is to be continued past the initial medical assessment, oxygen therapy is to be prescribed in writing on the patients National Inpatient Medication Chart. Documentation must include:

1. Mode of administration
2. Maximum flow rate and oxygen percentage (Especially important in the patient with known CO2 retention)
3. Target SpO2. Medical prescription for permissive hypoxemia (i.e. low SpO2 as accepted by the treating physician taking into account the patient's history, where normal range SpO2 or PaO2 range is unattainable) is necessary to differentiate treatment goals. Documentation to be attended in the patient's medical record and on the Prescribed Modifications to Adult PACE Calling Criteria.
4. Length of time oxygen is to be delivered.

ANALGESIA

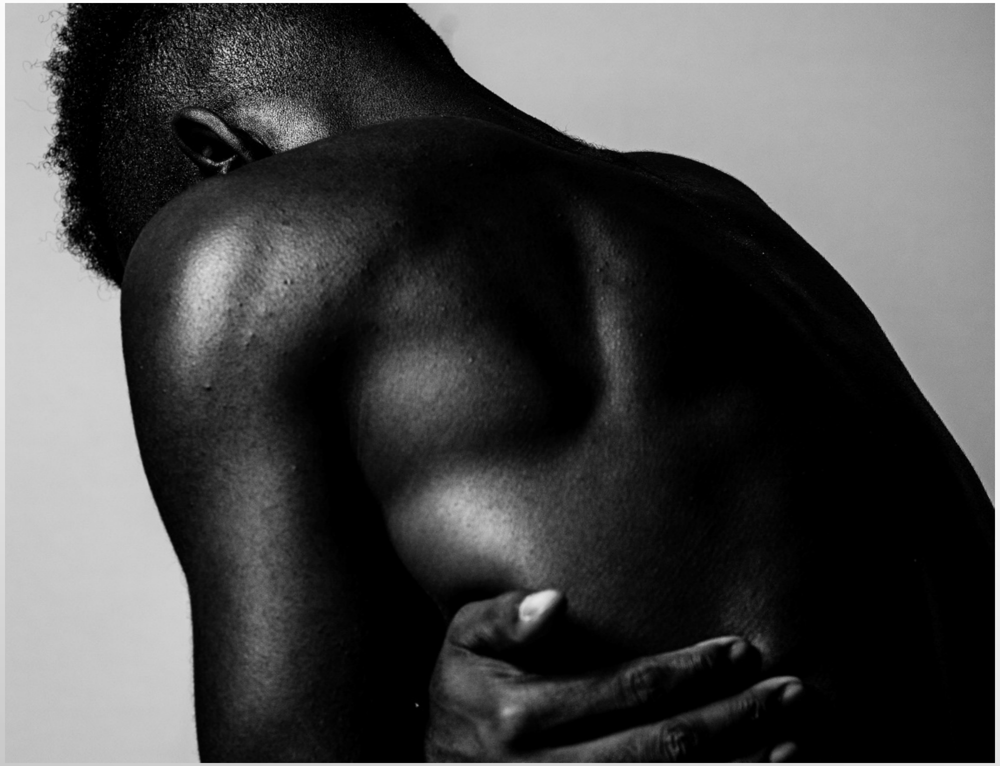

Photo by Sam Burriss on Unsplash

EARLY ANALGESIA LESS PNEUMONIA AND MORTALITY

EPIDURAL

- GOOD EVIDENCE FOR ≥3 RIB FRACTURES, AND ≥ 60YEARS, IMPROVES VENT. FUNCTION, PAIN, LOS, COMPLICATIONS
- PNEUMONIA 6X MORE LIKELY IN IV / MORTALITY 2X
- MAY CAUSE HYPOTENSION 20-100%, TECHNICAL

(BAKER & LEE, 2016; BULGER, EDWARDS, KLOTZ, & JURKOVICH, 2004; GAGE, RIVARA, WANG, JURKOVICH, & ARBABI, 2014; MCKENDY ET AL., 2017; WISNER, 1990; ZAW ET AL., 2015)

PARAVERTEBRAL BLOCK – AS EFFECTIVE AS EPIDURAL FOR PAIN AND VENTILATORY FUNCTION. LESS TECHNICAL

(KARMAKAR ET AL., 2003; MOHTA, OPHRII, SETHI, AGARWAL, & JAIN, 2013; SHUKLA, GHAFFAR, AUANG, RAJAH, & TAN, 2008)

SYSTEMIC

- COMBINATION THERAPY
- ORALS (E.G PARACETAMOL / NSAIDS) = REDUCED OPIOIDS
- TRANSDERMAL PATCHES DECREASED LOS, PAIN, NARCOTIC USE
- PCA REDUCED LOS 10%

HOW TO ACTIVATE AT SDMH

- CALL SURG REG AND ICU REG
- EMR WILL NOTIFY CRN, PAIN AND PHYSIO BUT YOU CAN CALL THEM AS WELL
- IF HAVEN'T ATTENDED WITHIN 60 MINUTES – PLEASE CALL
- IF A TRAUMA CALL/STANDBY WITH CHEST WALL INJURY FOR LOCAL ADMISSION PLEASE ACTIVATE CHIP

If urgent please phone relevant clinician/s. Please see below:

**Pain CNC:**	9370 (DECT)
**Pain Registrar:**	9460 (DECT) 0800 - 2100hrs
**Pain (Anaesthetics) A/H:**	VMO if needed via mobile - tba by ED Medical Officer
**Surgical Registrar:**	0411 025 497
**ASET:**	9563 (DECT)
**ICU Liaison/Outreach:**	9336 (DECT) or page 338
**ICU Registrar:**	9785 (DECT) or page 318
**ED Physio:**	9561 (DECT) Business Hours or 9459 (DECT) Weekends/Public Holidays

Note: *On weekends, new referrals must come via nursing supervisor as there is only one physio for the whole hospital so work will be prioritised.*
eMR referrals for inpatients during regular hours.

HOW TO ACTIVATE AT TWH

- CALL SWITCH (222)
- IF HAVEN'T ATTENDED WITHIN 60 MINUTES – PLEASE CALL
- IF A TRAUMA CALL/STANDBY WITH CHEST WALL INJURY FOR LOCAL ADMISSION PLEASE ACTIVATE CHIP

**TWH Hospital call 222**	
**Pain CNC**	185
**Trauma CNC**	380
**Pain Reg**	556
**Pain (Anaesthetics) after hours**	533
**Surg Reg**	398
**ASET**	390
**ICU Liaison**	120
**ICU Reg**	113
**Physio**	368
**Emergency CNC**	1220
**Bed manager**	109

- ED calls 222

- ED state ***"please activate CHIP MRN is 123456"***

- Switch activate group page 880
- **ED CHIP MRN 123456**

COMPLICATION PREVENTION

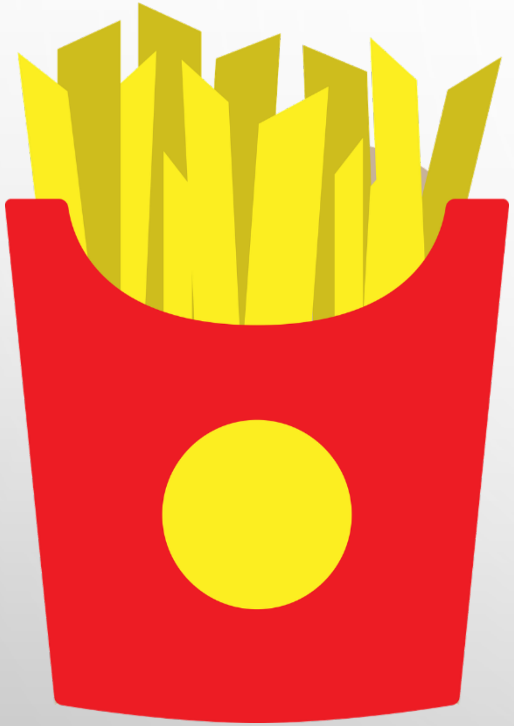

Image by Tomislav Kaučić from Pixabay

ED

- ASSESS, ANALGISE, REASSESS AND ACTIVATE
- IN CHARGE – MONITOR AND ACTIVATE
- ESCALATE AND ENSURE ATTENDANCE

WARD

- ADVOCATE, MONITOR
- IMPLEMENT CARE BUNDLE ++

EVERYWHERE

- ADEQUATE ANALGESIA TO DB&C
- EARLY MOBILISATION
- CALL ICU REG/LIAISON/CRN, PAIN TEAM, PHYSIO IF NEED BE
- APERIENTS
- PHYSIO ++
- INVOLVE FAMILY AND PATIENT
- HANDOUT HTTPS://WWW.ACI.HEALTH.NSW.GOV.AU/__DATA/ASSETS/PDF_FILE/0010/294337/FRACTURED_RIBS_PATIENT_FACTSHEET_2015.PDF

Patient Factsheet **Hospital:**

Fractured Ribs

Understanding fractured ribs

Rib fractures are one of the most common injuries to the chest. Ribs will usually fracture at the point of impact or towards the back, where they are weakest.

Typically, this causes pain on deep breathing and coughing, and tenderness over one or more ribs.

Chest X-rays may not show the broken rib but are useful to check for underlying lung injury. The findings may help to make the correct diagnosis.

There is potential for underlying organ injury when ribs are fractured; your doctor or health practitioner will assess you to exclude this.

Know the Facts

- Pain may get worse for a week and last for up to eight weeks. If you are discharged from the emergency department it is important that you understand the information your doctor gives you about pain relief medications.
- Older people, smokers, those with lung disease and people with multiple rib

Tips to help your recovery

- The most important thing is to get any pain under control. Breathing exercises will not be effective unless your pain is controlled.
- Take your pain relieving medications regularly, as prescribed by your doctor, and continue to speak with your local doctor or pharmacist about maintaining your pain relief. The medications should provide a good and constant level of pain control and avoid peaks of pain.
- Strenuous activities should be avoided for the first 3-4 weeks, after which physical activity may be recommenced as pain allows. If the pain is increasing you may be doing too much. Talk to your doctor or physiotherapist about this.
- Avoid contact sports for at least 6 weeks to prevent further damage, unless otherwise advised by your doctor or physiotherapist.

Fractured Ribs

Exercises to try

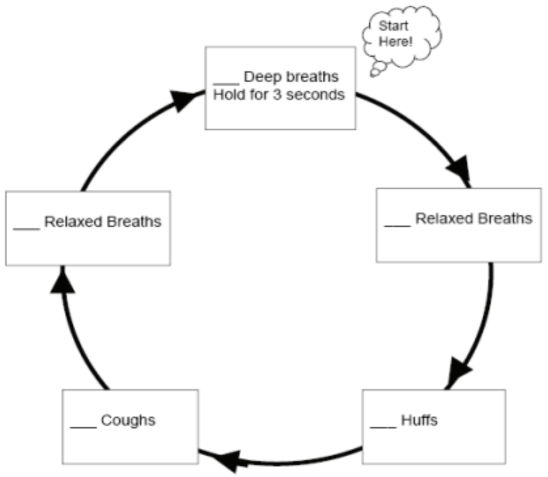

Repeat___ times
Every hour

- Try holding a cushion firmly against the painful site when you huff and cough to decrease the pain.
- Sit out of bed and keep moving as much as you feel comfortable. This will decrease the risk of developing lung complications.

Instructions:

____

Follow up treatment

- Physiotherapy follow up as directed by your doctor.
- See your local doctor if you have any concerns, uncontrolled or increasing pain, breathing problems, fever or develop a cough with sputum.

Seeking help:

[illegible]

AVAILABLE FROM ECI WEBSITE

HTTPS://YOUTU.BE/VLMZ1PJZMBK

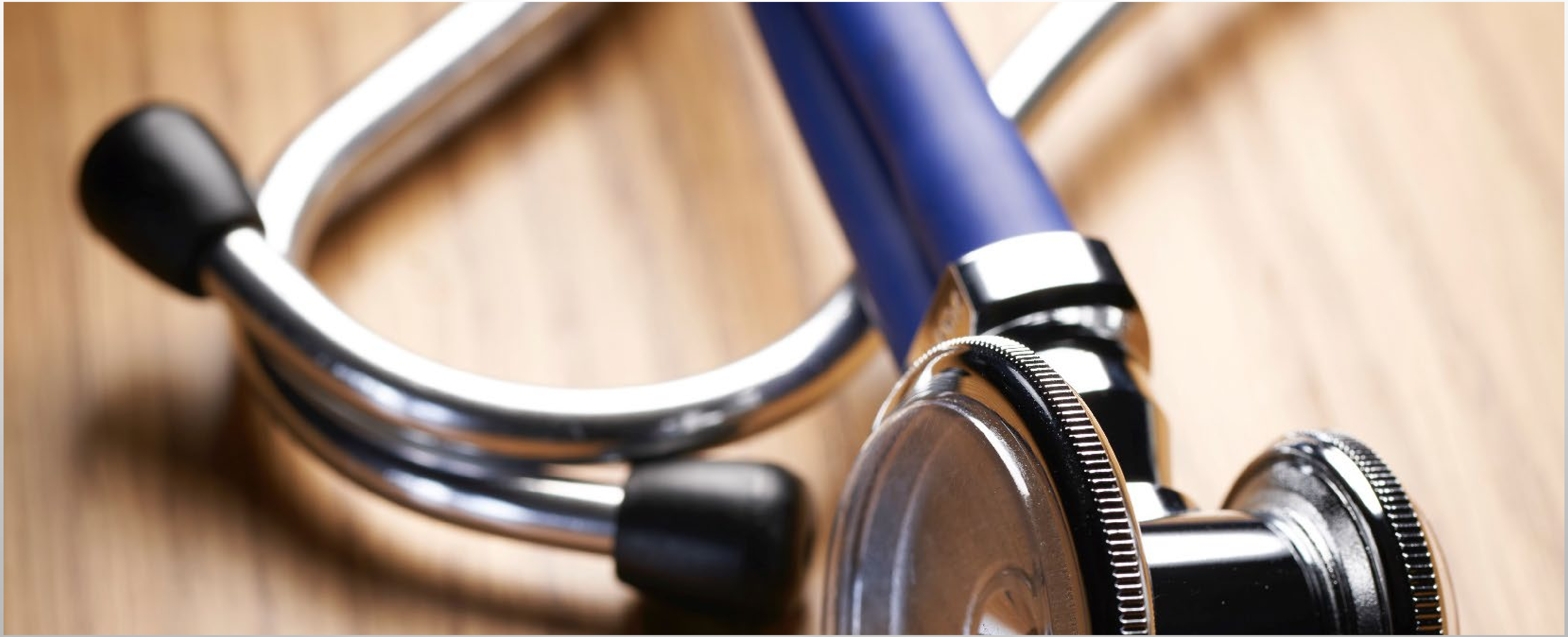

Photo by Bill Oxford on Unsplash
