# Supplementary material for: Impact of a care bundle for patients with blunt chest injury (ChIP): A multicentre controlled implementation evaluation
Source: PLoS One. 2021 Oct 7;16(10):e0256027. doi: 10.1371/journal.pone.0256027 (PMC8496821; doi:10.1371/journal.pone.0256027)
Supplement: S3 File — (PDF) [file pone.0256027.s003.pdf]

ICD-10-AM and DRG v6 codes for ChIP study

ICD-10-AM codes

ICD-10-AM codes for eligibility
S20.2 - Contusion of thorax
S20.30 - Other superficial injuries of front wall of thorax unspecified
S20.31 - Other superficial injuries of front wall of thorax abrasion
S20.38 - Other superficial injuries of front wall of thorax other
S20.41 - Other superficial injuries of back wall of thorax abrasion
S20.48 - Other superficial injuries of back wall of thorax other
S20.80 - Superficial injury of other and unspecified parts of thorax unspecified
S20.81 - Superficial injury of other and unspecified parts of thorax abrasion
S20.88 - Superficial injury of other and unspecified parts of thorax other
S22.2 - Fracture of sternum
S22.31 - Fracture of first rib
S22.32 - Fracture of one rib other than first rib
S22.40 - Multiple rib fractures unspecified
S22.41 - Multiple rib fractures involving first rib
S22.42 - Multiple rib fractures involving two ribs
S22.43 - Multiple rib fractures involving three ribs
S22.44 - Multiple rib fractures involving four or more ribs
S22.5 - Flail chest
S23.4 - Sprain and strain of ribs and sternum
S27.31 - Contusion and haematoma of lung
S27.81 - Injury of diaphragm
S27.88 - Injury of other specified intrathoracic organs and structures
S27.32 - Laceration of lung

*ICD-10 AM = International Statistical Classification of Diseases and Related Health Problems, Tenth Revision, Australian Modification

AR-DRG v 6 codes

	DRG_type	DRG description
801A	S	OR Procedures Unrelated to Principal Diagnosis W Catastrophic CC
E40B	O	Respiratory System Diagnosis W Ventilator Support W/O Catastrophic CC
E66A	M	Major Chest Trauma W Catastrophic CC
E75A	M	Other Respiratory System Diagnosis W Catastrophic CC
E66B	M	Major Chest Trauma W Severe or Moderate CC

E66C	M	Major Chest Trauma W/O CC
E75B	M	Other Respiratory System Diagnosis W Severe or Moderate CC
E75C	M	Other Respiratory System Diagnosis W/O CC
E01B	S	Major Chest Procedures W/O Catastrophic CC
E02C	S	Other Respiratory System OR Procedures W/O CC
E40A	O	Respiratory System Diagnosis W Ventilator Support W Catastrophic CC
E41Z	O	Respiratory System Diagnosis W Non-Invasive Ventilation
E67A		Respiratory Signs and Symptoms W Catastrophic or Severe CC

*AR-DRG = Australian Refined Diagnosis Related Groups
