# Supplementary material for: Impact of a care bundle for patients with blunt chest injury (ChIP): A multicentre controlled implementation evaluation
Source: PLoS One. 2021 Oct 7;16(10):e0256027. doi: 10.1371/journal.pone.0256027 (PMC8496821; doi:10.1371/journal.pone.0256027)

Additional file 4.

Figure 1. High Flow Nasal Prongs pre and post ChIP implementation by Treatment groups [for all patients] – Quarterly

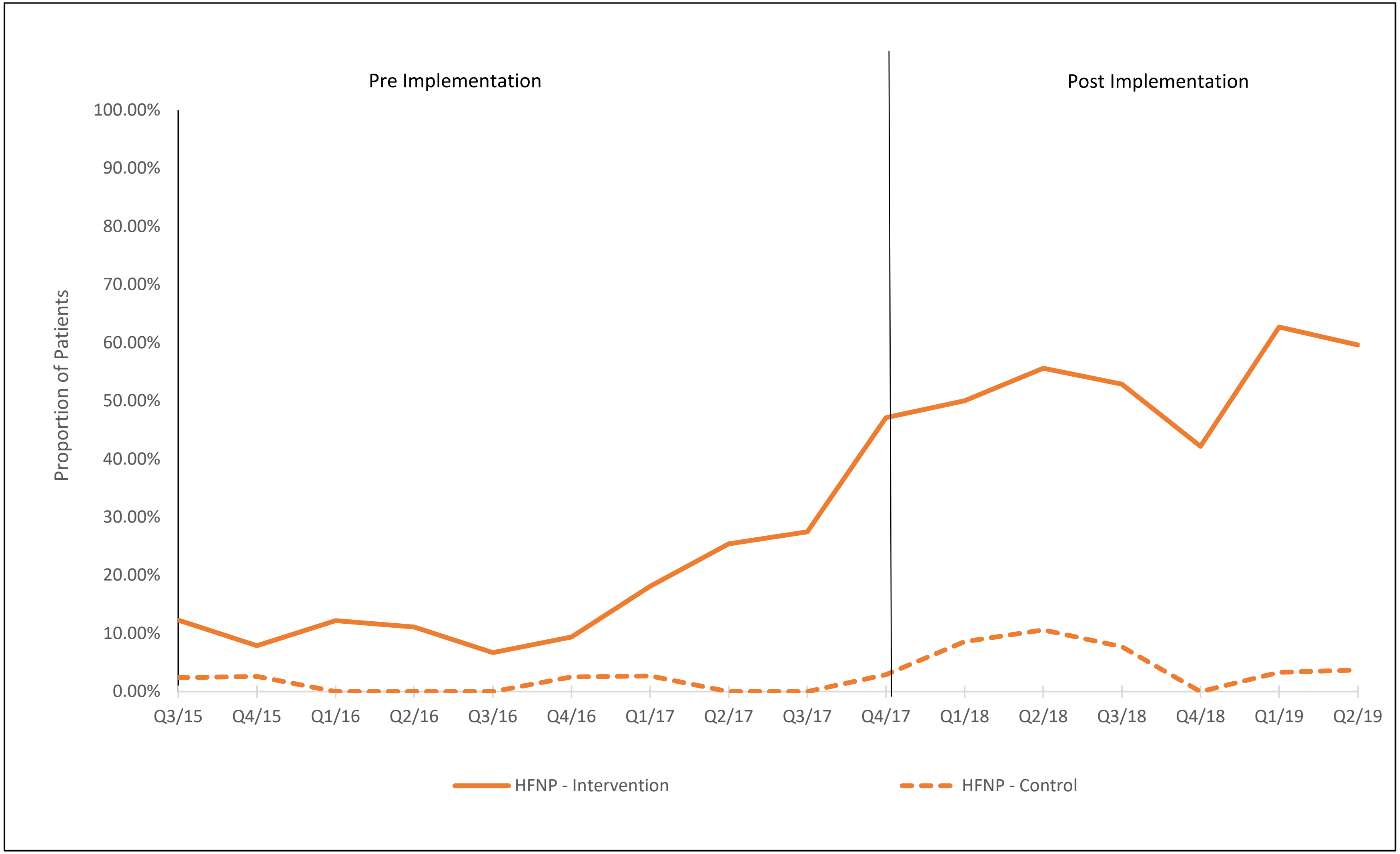

Figure 2. Analgesia within 24 hours pre and post ChIP implementation by Treatment groups [for all patients] – Quarterly

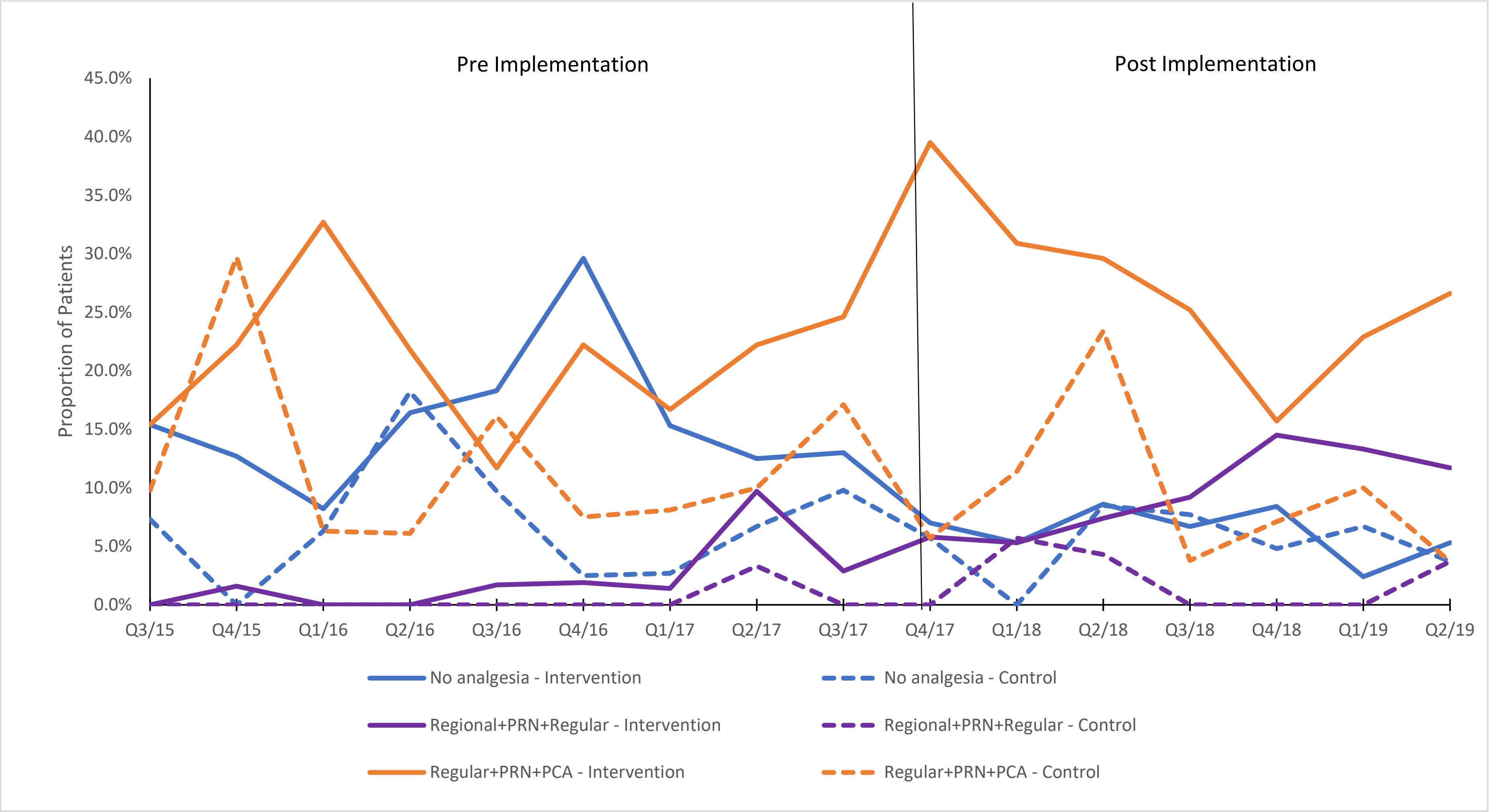

Supplement: S6 File — (PDF) [file pone.0256027.s006.pdf]
